# The hospice as a learning space: a death education intervention with a group of adolescents

**DOI:** 10.1186/s12904-021-00747-w

**Published:** 2021-04-07

**Authors:** Ines Testoni, Lorenza Palazzo, Lucia Ronconi, Stefania Donna, Paolo Francesco Cottone, Michael Alexander Wieser

**Affiliations:** 1 Department of Philosophy, Sociology, Pedagogy and Applied Psychology (FISPPA), University of Padova, Via Venezia 14, 35131 Padova (PD), Italy; 2 Emili Sagol Creative Arts Therapies Research Center, University of Haifa, 3498838 Haifa, Israel; 3 Department of Psychology, University of Klagenfurt, 9020 Klagenfurt am Woerthersee, Austria

**Keywords:** Death education, Adolescents, Alexithymia, Mixed methods, Denial of death

## Abstract

**Background:**

The denial of death in Western society deprives young people of the tools to derive meaning from experiences of death and dying. Literature shows that death education may allow them to become familiar with this topic without causing negative effects. This article describes the effects of a death education course with adolescents, wherein participants were given the opportunity to meet palliative doctors and palliative psychologists at school and in a hospice, where they were able to converse with the families of the dying.

**Methods:**

This study used mixed methods and included an evaluation of a death education intervention with longitudinal follow-up of outcomes. The course involved 87 secondary school students (experimental group) aged between 16 and 20 years. We also recruited a control group of 76 similarly-aged students to observe differences. The variables we examined were: alexithymia, representation of death, value attributed to life and spirituality. These were measured with the following instruments: the Toronto Alexithymia Scale-20, the Testoni Death Representation Scale, the Personal Meaning Profile and the Spiritual Orientation Inventory, respectively. To better understand how the students perceived the experience, we asked the experimental group to answer some open-ended questions. Their answers were analysed through thematic analysis.

**Results:**

The study showed that death education and the hospice experience did not produce negative effects, but rather allowed students to decrease alexithymia, improving their ability to recognise and express emotions. Thematic analysis revealed that all participants perceived the experience as very positive.

**Conclusions:**

Our findings affirm that death education programs can be successfully implemented in high schools, and that they can usefully involve local hospices and palliative care professionals, especially physicians and psychologists.

**Supplementary Information:**

The online version contains supplementary material available at 10.1186/s12904-021-00747-w.

## Background

The denial of death and dying characterises contemporary Western culture [1]. Mourning is no longer a social process, but something that must be concealed and suffered in solitude [2–4]. This is why young people are deprived of opportunities to emotionally experience death and dying prior to an actual experience of loss, and why they struggle to attribute an existential meaning to death.

Existing literature shows that a mature concept of death develops in accordance with one’s developmental age [5–8]. The representation of death in adolescence is halfway between the magical thinking of childhood and the complex ideation of adulthood [9, 10], and it is characterised by the maturation of abstract cognitive thinking skills, which open up existential questions about life and the afterlife and enable reflection on the meaning of death. This is the period in which personal opinions on religion develop in parallel with trust or estrangement from one’s childhood faith, and in which one begins to search for their own spirituality and transcendent truth [7, 11–13]. The spiritual dimension concerns all individuals, as it entails a fundamentally human aspiration for transcendence, which improves one’s connection with others, with nature and with the divine. It is expressed through multiple modes, beliefs, symbols, attitudes and moral and ethical values [14]. Several studies have shown that it is important to stimulate this dimension in adolescence because it protects against depressive symptoms and other mental health risks [15–17] and improves resilience when dealing with negative life events [18–20].

Death Education (DE) may help adolescents to manage some of the issues inherent to death and dying, despite the current cultural denial of death [21]. Through a multidisciplinary approach, DE stimulates reflection on topics related to death and dying, helping participants to create a personal existential meaning for this phenomenon. This is especially useful for adolescents in this ‘death-free generation’, who have never had the opportunity to accompany dying relatives in their last moments of life, in contrast to those in previous centuries [22, 23]. DE exercises help to normalise the concept of mortality without causing negative effects [24–27]. Existing literature highlights how important it is to recover deep thoughts on the meaning of existence and finitude, as these enable children to better manage the anxiety of death, to create a dialogue on the theme of dying and to improve their ability to deal with this issue [22, 28–33]. These interventions increase peer social support and help individuals to make sense of negative experiences, become more emotionally expressive and listen without judging the experiences of others, thereby increasing empathic understanding [34–37]. Moreover, they help to clarify one’s representation of death, making it more mature by reducing the anxiety caused by fantastic or distorted infantile representations [38, 39] and by furnishing it with mature language that can adequately express death experiences [32, 40]. In particular, DE may reduce alexithymia - that is, the inability to recognize and verbalize one’s own emotions and those of others and to communicate them to others [4, 41] - because it allows individuals to recognise and give voice to negative feelings [4, 29, 32, 42]. Previous literature that compared the verbal and artistic productions of DE participants with those of their peers revealed that, thanks to the exploration of existential anguish that it enables, DE improves people’s ability to give voice to loss and creates a corresponding emotional vocabulary [4, 43, 44].

Despite ample evidence of DE’s positive effects for adults, there is little literature on DE’s utility for adolescents, especially when palliative care is involved in the course [4, 27, 45]. The present article fills this gap by presenting a study on a DE course with adolescents that involved a local hospice, configured as a space for learning about life’s limits and the possibility of transcendence.

## Methods

### Aims

Because the literature shows that DE helps adolescents better manage their emotions, especially the negative ones caused by the fear of death, the first aim of this study was to show that an experience of DE involving one hospice and its professionals may help students reduce alexithymia. In particular, since adolescence is a time when emotions may be very confusing, the challenge of the study was to confirm that the hospice environment can be helpful in DE courses and that it can allow for a deep exploration of issues related to death. In fact, it was hypothesized that the reaction to the hospice experience would not result in negative consequences, but instead it was positive and increased participants’ ability to reflect on meaning of life and the sense of limit, without anxiety.

### Activities and participants

The DE course lasted 2 months. Two psychologists conducted and supervised all the activities, some of which also included professionals from other fields. Formal activities included (1) lectures on the themes of death and dying in different cultures and religions, conducted by a monk and anthropologist who specialises in the accompaniment of the dying; and (2) lectures on palliative care and the work of professionals in the end-of-life field, conducted by one palliative and one paediatric doctor and two palliative psychologists. Informal activities included meditation, writing and sharing personal epitaphs, viewing excerpts from films and documentaries on the theme of death, meeting with mourners in support groups (organised through the hospice), and photovoice exercises where participants shared photos of things that they associated with loss and the afterlife. After this preparation, students visited the local hospice to see how it was organised and to meet palliative professionals. Finally, they met and conversed with dying patients’ relatives.

The participants were third-, fourth- and fifth-year students from two secondary schools in a Northern Italian town who had taken part in a similar project the previous year and were motivated to participate for the following year [4]. The experimental group was not randomised because only certain classes volunteered to participate in DE. The control group was also not randomized because it was formed with classes of students from the same schools and the same age in which professors and students did not want to take part in the project. Of the 180 students that participated in the overall study (including the control group), only 163 completed the entire survey: 87 in the experimental group (DE group) and 76 in the control group (NoDE group). Sex distribution was not consistent: males were 33% of the total participants; females were 81% in the DE group and 53% in the NoDE group. The average age of all the students was 17.5 years: in particular, the students’ age of DE group was between 16 and 20 years old (Mean = 17.06; *SD* = 0.932) and the students’ age of the NoDE group was also between 16 and 20 years old (Mean = 18.03; *SD* = 0.938, range: 16–20 years). No significant differences were found between the two groups with respect to religiosity (believers: 41% DE and 47% NoDE), active participation in religious practices (participants: 83% DE and 74% NoDE) or socio-demographic variables (Table 1). Participants were informed about the objectives and procedures of the study. Their participation was voluntary and the confidentiality of their responses was guaranteed. Written informed consent was obtained from all students over 18 years old and from both parents for students aged 16 and 17 years old. The study followed the ethical principles of the Declaration of Helsinki and the Code of Conduct of the American Psychological Association (APA); moreover, it was approved by the Ethics Committee of the University of Padua (protocol number: 1132CC044320CF24685EFECF59575C22).

**Table 1:** Participants’ demographic characteristics

	DE group (***N*** = 87)			No DE group (***N*** = 76)	
	Frequency		Percentage	Frequency	Percentage
Sex	M	17	19.5%	36	47.4%
	F	70	80.5%	40	52.6%
Believe in God	Yes	36	41.4%	36	47.4%
	No	51	58.6%	40	52.6%
Participation in religious practices	Yes	15	17.2%	20	26.3%
	No	72	82.8%	56	73.7%
Age	Mean (SD)	17.06 (0.93)		18.03 (0.94)	

### Instruments and analysis methods

The project involved a mixed methods design, which allowed us to investigate the different elements of the project with the most suitable tools [46, 47]. We used a questionnaire with both Likert-scale and open-ended questions that measured participants’ age, sex, religious beliefs and participation in religious practices. These questions were supplemented with those from the following scales:

The *Testoni Death Representation Scale* (TDRS) is a questionnaire composed of six items that are scored using a 5-point Likert scale, wherein 1 is ‘totally disagree’ and 5 is ‘totally agree’. This scale assesses both philosophical and psychological dimensions, and it is the first tool to evaluate the ontological representations of death by arranging them along a continuum, from ‘death as total annihilation’ to ‘death as a passage’ into some form of afterlife. This tool has also been used in previous studies of DE, which examined the relationship between religiosity, spirituality, death, well-being [4, 29, 41], suffering and palliative care [40, 48].

The *Toronto Alexithymia Scale-20* (TAS-20) [42] is a self-report tool that assesses alexithymia, a personality construct characterised by the inability to distinguish or describe emotions in oneself or others. The TAS-20 is composed of 20 items that are measured on a 5-point Likert scale, and these are divided into three groups related to the following factors: (1) ‘ability to identify and recognise one’s own and others’ feelings’ (TAS-F1), (2) ‘competence to communicate and express one’s feelings to other people’ (TAS-F2) and (3) ‘externally oriented thinking’ (TAS-F3), which is the tendency to reflect one’s affects outwards (e.g., ‘others ask me to talk more about my feelings’) [42, 49, 50]. The TAS-20 has been translated and validated for use with Italian populations, and this version exhibits good validity for both clinical and non-clinical groups [51, 52].

The *Personal Meaning Profile* (PMP) [53] evaluates the personal meaning attributed to one’s life. The self-report questionnaire is composed of 57 items scored on a 7-point Likert scale, wherein 1 represents ‘not at all’ and 7 represents ‘very’. The items are divided into seven subscales that measure the dimensions of meaning in life: Achievement (ACH), with 16 items (e.g., ‘I am able to use my abilities to the fullest’); Relationship (RLT), with nine items (e.g., ‘I have trusted people to give me emotional support’); Religion (RLG), with nine items (e.g., ‘I believe that there is an order and an end in the universe’); Self-Transcendence (ST), with eight items (e.g., ‘I seek higher values that exceed personal interests’); Self-Acceptance (SA), with six items (e.g., ‘I have learned to live with suffering, getting the best out of it’); Intimacy (INT), with five items (e.g., ‘I have someone to share personal emotions with’); and Fair Treatment or Perceived Justice (FT), with four items (e.g., ‘I have received the right portion of opportunities and rewards’). We used the Italian translation of the questionnaire, which confirmed the structure and reliability of the seven items [4].

The *Spiritual Orientation Inventory* (SOI) by Elkins et al. [54] includes eight items that investigate the extent to which an individual relies on a transcendental dimension, understood as a human phenomenon that is not necessarily related to religion. The religious dimension, in fact, refers more to individuals’ social encounter with the divine [54, 55]. Respondents evaluate their agreement on a 5-point Likert scale, wherein 1 corresponds to ‘totally disagree’ and 5 to ‘totally agree’. We used a previous Italian use of the questionnaire, which has been used in a study concerning the relationship between spirituality and representation of death and that confirmed the structure and reliability of the scale [56].

After the DE course, the DE group answered two additional open-ended questions related to the experience: ‘What impressed you most about this experience?’ and ‘Do you feel, after the course and the experience in the hospice, that you can deal with death in a different way? If yes, why? If not, why not?’ These questions were designed to uncover the most salient aspects of the course and its effectiveness in terms of modifying death representations, respectively. Because the NoDE group did not complete the DE course, they did not respond to these questions.

These instruments and open-ended questions were chosen based on the literature related to DE, in which DE has been shown to help better manage emotions and in particular the fear of death [4, 27]. In the present study, spirituality was also considered to detect its interaction with alexithymia, as already done in other studies [13, 41]. Mixed method research design was chosen to better investigate these interactions.

We verified the reliability of the four questionnaires by calculating their Cronbach’s alpha coefficients. We used the t-test for independent samples to determine whether there were initial differences between the DE group and the NoDE group at Time 1. Correlations between the questionnaires at Time 1 and between participants’ responses and social variables were evaluated using Pearson’s correlation coefficients. We evaluated the effects of the DE course – i.e., the changes exhibited by the DE group relative to the NoDE group over time – by performing a two-way analysis of variance (ANOVA) with the following factorial design: time (pre-test and post-test) x group (DE and NoDE) for each variable under study. The analyses were performed with the Statistical Package for Social Science (SPSS) statistical analysis software [57].

We used thematic analysis to interpret the qualitative responses, which allowed us to recognise prevailing meanings and concepts [58]. Specifically, two procedures were used: bottom-up for the first question, wherein categories were created based solely on the participants’ responses, without any a priori code; top-down for the other questions, using ontological representations of death as a priori categories [59, 60]. The process involved six phases: preparatory organisation, creating categories or themes, coding data, understanding the textual data, searching for alternative explanations and drafting the report [61]. We coded the open-ended questions using the qualitative analysis software ATLAS.ti [62] which allowed us to apply the codes to the text parts and join them into families of codes from which the prevalent themes emerged. We also created graphs to describe the structure of the categories and the logical relationships between responses.

## Results

### Quantitative analysis results

The analysis showed that all the given instruments have generally good reliability, both for Time 1 (pre-test) and Time 2 (post-test) (TDRS α_pre = .81, α_post = .82; TAS-20 total α_pre = .79, α_post = .82; PMP total α_pre = .93, α_post = .93; SOI α_pre = .70, α_post = .68). The lowest reliability scores were found in the TAS-F3 at Time 2 (α = .54) and in the FT subscale of the PMP at Times 1 and 2 (α_pre = .53, α_post = .54).

We used the t-test for independent samples to determine whether there were initial differences between the DE group and the NoDE group at Time 1. The only significant result pertains to the spirituality dimension, which was higher among participants in the DE group than among those in the NoDE group (*M* = 3.40, *SD* = 0.61 for the DE group and *M* = 3.21, *SD* = 0.65 for the NoDE group; *t* = 2.01, *df* = 161, *p* = .047). The other instruments did not show significant differences between the two groups.

The correlations between all the instruments are presented in Table 2. A higher score in the TDRS, which corresponds to a representation of death as annihilation, correlates negatively with total PMP, the RLG scale of the PMP and the spirituality score of the SOI. Total alexithymia correlates negatively with total PMP and all its subscales. More specifically, higher scores in TAS-F1 and TAS-F2 are associated with greater spirituality and lower scores in total PMP and some of its subscales, while higher scores in TAS-F3 are associated with lower PMP scores and a lower score in spirituality. A higher total PMP ​​is associated with less alexithymia, greater spirituality and representations of death as a passage. Finally, spirituality positively correlates with the RLG scale of the PMP.

**Table 2:** Correlations between measures

Correlations														
	1	2	3	4	5	6	7	8	9	10	11	12	13	14
**1. TDRS**														
**2. TAS_Tot**	0.13													
**3. Factor 1**	0.04	0.79**												
**4. Factor 2**	0.10	0.80**	0.55**											
**5. Factor 3**	0.13	0.50**	− 0.01	0.17*										
**6. PMP_Tot**	−0.23**	−0.58**	− 0.46**	− 0.47**	−0.28**									
**7. ACH**	0.05	−0.47**	−0.42**	− 0.35**	− 0.19*	0.76**								
**8. RLT**	−0.06	−0.52**	−0.40**	− 0.44**	−0.25**	0.68**	0.48**							
**9. RLG**	−0.57**	−0.16*	−0.05	− 0.10	−0.20**	0.55**	0.25**	0.15						
**10. ST**	−0.08	−0.42**	−0.33**	− 0.29**	−0.27**	0.76**	0.80**	0.38**	0.42**					
**11. SA**	−0.11	−0.36**	−0.46**	− 0.30**	0.05	0.60**	0.33**	0.41**	0.17*	0.27**				
**12. INT**	−0.10	−0.36**	−0.15	− 0.40**	−0.26**	0.60**	0.28**	0.42**	0.20**	0.27**	0.18*			
**13. FT**	−0.08	−0.42**	−0.37**	− 0.34**	−0.16*	0.70**	0.46**	0.45**	0.22**	0.41**	0.45**	0.33**		
**14. SOI**	−0.36**	0.06	0.23**	0.16*	−0.32**	0.15*	−0.08	0.03	0.51**	0.14	−0.05	0.07	0.01	

*p < .05; **p < .01

Only the TDRS showed a significant correlation with age (*r* = .18, *p* = .020), highlighting the fact that older adolescents are more likely to represent death as annihilation than younger adolescents. Sex differences were observed in the TAS-20, both in total score and in its three factors. Females scored always higher than males except for externally oriented thinking factor where males scored higher than females (see supplementary material Table A). Moreover, sex differences were observed in two subscales of the PMP: Achievement (ACH), and Self-Transcendence (ST). Females scored lower than males in both subscales (see supplementary material Table A).

As expected, differences between believers and non-believers in God were found in the TDRS, the RLG subscale of the PMP and the SOI. Believers in God scored lower on death as annihilation than non-believers and higher on the Religion (RLG) subscale of the PMP (*M* = and on the Spiritual Orientation Inventory (SOI) than non-believers (see supplementary material Table B).

We observed a significant interaction between time and group – that is, a different trend in time for the DE group compared to the NoDE group – for total TAS-20 (*F* (1,161) = 3.98, *p* = .048) and TAS-F1 (*F* (1,161) = 7.22, *p* = .008). In particular, we recorded a significant reduction in mean scores for alexithymia from pre-test to post-test in the DE group (*t* = 2.34, *df* = 86, *p* = .020 for TAS-20 total; *t* = 3.46, *df* = 86, *p* = .001 for TAS-F1), while the students in the NoDE group showed a constant mean score over time (*t* = − 0.54, *df* = 75, *p* = .588 for TAS-20 total; *t* = − 0.44, *df* = 75, *p* = .659 for TAS-F1). The other variables did not show significant changes, but rather general stability. In other words, the results showed the stability of death representations, meanings attributed to life and spirituality, highlighting that the DE course did not produce negative effects. Rather, scores for total TAS-20 and TAS-F1, which refers to an individual’s ability to identify and recognise their feelings and those of others, decreased from Time 1 to Time 2, confirming the third aim as well as the study instruments’ ability to capture differences between adolescents at different phases of the test’s administration (see Table 3).

**Table 3:** Means and standard deviations of the scores to the pre and post intervention questionnaires of DE group and No DE group

Scale	DE group (***N*** = 87)				No DE group (***N*** = 76)			
	pre-test		post-test		pre-test		post-test	
	M	SD	M	SD	M	SD	M	SD
TDRS	3.20	0.93	3.13	0.89	3.34	0.93	3.31	0.87
TAS_Tot	51.65	9.38	49.79	10.38	50.36	12.44	50.82	11.63
Factor 1	2.68	0.79	2.44	0.80	2.49	0.97	2.52	0.85
Factor 2	3.03	0.83	2.93	0.91	2.86	1.01	2.82	0.97
Factor 3	2.22	0.58	2.26	0.54	2.33	0.59	2.38	0.50
PMP_Tot	4.57	0.65	4.49	0.73	4.62	0.72	4.65	0.64
ACH	4.94	0.94	4.91	0.98	5.03	0.96	5.00	0.90
RLT	5.04	0.79	4.93	0.92	5.06	1.01	5.11	0.88
RLG	3.71	1.20	3.62	1.36	3.57	1.19	3.69	1.30
ST	4.44	1.04	4.46	1.11	4.52	1.06	4.57	0.94
SA	4.30	1.15	4.25	1.13	4.54	0.89	4.61	0.78
INT	4.75	1.14	4.61	1.20	4.80	1.29	4.85	1.13
FT	4.76	0.92	4.61	0.99	4.81	0.98	4.74	0.93
SOI	3.40	0.61	3.34	0.63	3.21	0.65	3.27	0.61

### Qualitative analysis results

Two main themes emerged from participants’ responses to the open-ended questions: (1) appreciation for the DE experience, and (2) contemplating death-related issues helps to improve meaning-making concerning life and the future. The numbers indicated in the following quotes specify the interview number of each participant (1 to 87) and the order number that refers to the quote on the working text in Atlas.ti. The names are fictitious to respect the anonymity of participants.

### First main theme: appreciation for the DE experience

Participants specified which activities they most appreciated during the course. These were mainly the experiential activities and the meetings with both external guests and hospice professionals. Marta, for example, said:

“The experience I liked the most was the visit to the hospice because I discovered that there are people who accompany the terminally ill until the moment of death, and this impressed me a lot. I really appreciated the spirit of collaboration that exists between young and old workers and the way they face death. Through their stories, I found that being in contact with it every day does not lead to depression or isolation” (11:2).

Again, with respect to the hospice, Alice reported:

“I was impressed when we went to the hospice, a place that was unknown to me until then. For me, being there was really a very important experience. I discovered a “new world”; it opened my eyes to a reality that I did not consider at all before” (44,1).

Instead Maria shared:

“I was very impressed by the testimony of the girl who told us about when her mother died. The meeting with her was a bit shocking, in the sense that it made me think about the attitude that a person takes towards such a painful situation. The experience in the hospice was the most important part of this course” (22:1).

Similarly, Claudio affirmed:

“I was very impressed by the hospice visit; it was touching, and it made me think a lot. The thing that intrigued me most about this experience was the vitality of Sister Anna. Her great sensitivity struck me deeply, and I thought for a long time about her ability to offer help to dying people” (66:2).

Instead, Lucia reported:

“I particularly liked the lesson with the monk and his knowledge of other religions and cultures, particularly Buddhism. I didn’t know anything about this perspective. It was really interesting” (39:1).

The experience was also appreciated by all the participants because it provided a protected place where they could open up and listen to the experiences of others. For example, Antonio said:

“I was very grateful to speak out loud about my problems and my emotions and to be able to express myself freely, without fear of being judged by others. I was able to talk about death and the fear it arouses in me without feeling ashamed” (81:1).

Many students appreciated the spirit of sharing and openness that they noticed among their classmates, and which they would not have otherwise expected. Paola shared:

“I was amazed at how many of my classmates were able to cooperate with each other and to open up to others, even though, sometimes, it might not have been easy. The experience in the hospice helped a lot with this” (70:3).

As reported by almost all the students, the professionalism and humanity of the experts facilitated all the educative processes. Sibilla wrote:

“I was struck by the character of the people who guided us in this experience, as I perceived their sensitivity and sweetness. In particular, the hospice professionals impressed me with their sensitivity. Moreover, I liked the way they managed to naturalise death, making it a natural thing” (59:3).

Finally, participants affirmed that they were grateful for the opportunity to reflect on themselves and on their deep emotions, as Elio explained:

“This experience helped me to get to know myself better and [taught me] to listen to myself more. The activities succeeded in bringing out the emotions present in each of us” (12.1).

For some, the course reassured them of their ability to ask for help during the elaboration of mourning. Moreover, the opportunity to talk about death and dying allowed some participants to face topics that they had never discussed before. Luigina declared:

“It was a useful experience that desensitised a topic that, unfortunately, is considered taboo and that nobody seems to face seriously” (72:2).

Some participants found positive aspects to the negative event of death, like Andrea, who said:

“It gave me the opportunity to reflect more on the meaning of life and death. I realised that, even in pain, you can find a glimmer of light, despite the difficulties” (87:2).

### Second main theme: contemplating death-related issues helps to improve meaning-making concerning life and the future

Almost all the students reported a positive change in their perspectives on life after learning about grief work and how to manage the end of life. Fiorenza affirmed:

“This course has allowed me to see death in a more serene way, as a natural event of life. It helped me develop a positive sense of death, not only seeing the negative. Thinking of it as a necessary but also natural thing helped me to appreciate the value and the meaning of life” (37:2).

She also reported a new perspective on the future, anticipating that she will have a greater ability to deal with this theme:

“I believe that I will be able to deal with the theme of death differently because the course showed me various facets of this event. Now I can think about my future with the awareness that it will finish, and that, sooner or later, it will happen, and I have to prepare for this to manage the suffering” (37:3).

Similarly, Gianfranco affirmed:

“Death exists and we will all have to face it. Now I see it more as a natural thing that has to happen, not as a sad thing. It is part of life. It just makes me a little sad to think about what I lost and can never get back” (15:2).

Some students, like Bruno, declared:

“Before, I saw death as total annihilation; death for me was always the end of everything, something that caused me so much fear. I would not know how to react in the face of grief” (23:1).

Although the course changed their initial perceptions about death, some participants said that they still fear losing any loved ones. For example, Matteo said:

“The course has made me change my vision about death, but I continue to be afraid of losing someone I love. I cannot manage this thought yet” (53:1).

However, other students, like Giuseppe, affirmed that:

“The experience in the hospice permitted me to understand that, in the future, when I’m very sick, I can ask experts in palliative care for help, and they will help me to manage my suffering. This permitted me to think of death in a different way, with less fear”.

In the same way, Matteo said:

“Through this course, I learned that I could ask for help after a loss to better process the grief, and this reassures me”.

The spiritual dimension was particularly appreciated, as Luigi affirmed:

“Thanks to these meetings, I was able to understand that death is not the end of everything. I feel that it is much more a passage than an inevitable end. I want to think of it more expansively” (61:4).

Instead, Francesca said:

“Now I consider death as the beginning of a new great adventure, much like how, at birth, the world of the child in the belly of the mother ends, and this is only the beginning of life” (40:2).

For some, the course confirmed and reinforced their vision of death as a passage, like for Luisella that said:

“I was never afraid of this passage. I no longer see it as something negative, but religion had already taught me this, and, after the course, it was confirmed” (62:2).

## Discussion

The current study has confirmed the first aim – that is DE course can help adolescents to better express their emotions by decreasing their level of alexithymia. We observed an improvement in alexithymia, as scores for this condition – namely, total alexithymia and TAS-F1 – decreased in the DE group but not in the NoDE group. From the thematic analysis, we observed that the participants reported not only the intensity of the emotions that were aroused through the course, but also the fact that they learned to reflect on these emotions, on themselves and on others. This corroborates the findings of previous studies, which showed that talking about one’s feelings and emotions in the context of DE improves participants’ expression and increases their emotional vocabulary [4, 27, 29, 32, 43, 45]. The moments of sharing that the course enabled made it possible to face the taboo of death. Indeed, some young people cited this as the key contribution of the course, as they had never had the opportunity to address this topic, either theoretically or personally, or to develop competency, confirming the presiding tendency to deny death [63, 64]. Reflecting on existential issues related to the end of life and creating a personal meaning around them [5–8, 17, 64, 65] allowed adolescents to not only familiarise, normalise and critically understand these topics, but also to think about some fundamental concepts that characterise death, such as universality and irreversibility. The participants also reported a greater ability to give meaning to death and pain, finding positive connotations despite the difficulties.

It was confirmed that the involvement of hospices in DE courses permits students to become familiar with this environment. In fact the students particularly appreciated the meeting with the hospice professionals, as well as their sensitivity and vitality, and this experience helped them to conceive of a new reality, abandoning the morbid stereotypes often linked to the hospice and its staff [45, 66, 67]. xxx.

Finally, with regard to the third aim, we observed that a DE course conducted by professionals in a school environment, despite eliciting death anxiety, does not produce negative effects in students. The results of our quantitative analysis showed that most of the psychometric traits we measured remained stable over time (between the pre-test and post-test phases), including those targeted in the DE group: representation of death, meanings attributed to life and spirituality. These results confirm previous studies that evaluated how DE increased death anxiety in order to eventually control it, without generating negative consequences [4, 13, 24–26, 29, 65, 68]. This contradicts the common opinion of many adults, who assume that it is inappropriate to expose adolescents to such heavy topics because it could raise too many concerns [69].

Moreover, we did not observe a change in questionnaire scores that would suggest a change in representations of death as total annihilation to death as a passage. Instead, we found that the score remained consistent from the pre-test to the post-test. However, in the responses to the open-ended questions, some participants reported that they had a nihilistic and materialistic conception of death prior to the DE course, understanding it as the end of everything – both body and spirit. After the course, they considered the possibility of a partially impermanent, de-subjectivized identity principle, thus conceiving an eternalist perspective compatible with the notion of death as a passage. This result confirmed those of previous studies [4, 25–27, 29, 32, 41, 44, 64, 70, 71], which demonstrated that reflecting on issues of finitude, mortality, loss and death encourages critical thought on transcendental spirituality, including questions about what exists after death, thereby diminishing the representation of death as annihilation. In line with previous studies, we also found that those who conceive of death as annihilation exhibit less spirituality and attribute lower value to their lives, and the literature indicates that this is true independent of the DE course [13, 31, 72]. Moreover, in line with the literature and, in particular, with what has been stated by Terror Management Theory [21, 64], we found that a representation of death as total annihilation correlates with less belief in religion and less participation in religious practices.

In sum, DE programs allow us to give voice to adolescents’ emotions about death by addressing the phenomenon of the ‘black hole’ [4, 27, 29] – the wall of silence and denial that surrounds the themes of death and dying – and to restore awareness to this important topic.

## Conclusions

The study confirmed that DE does not produce negative consequences, but rather promotes existential reflection, as evidenced by previous literature [4, 13, 25–27, 41, 44, 45]. We found that psychometric scores generally remained stable over time – namely those relating to ontological representations of death, personal meaning attributed to life and spirituality. Instead, alexithymia scores decreased in the DE group, suggesting that the DE project improved these students’ ability to recognise and express their own emotions and those of others.

The students appreciated the different experiential and theoretical activities in the course and the warmth, humanity and competence conveyed by the hospice professionals and guests who held the meetings. Students highlighted the particular importance of the hospice experience, which reassured them about their capacity to manage the end of life. This protected space offered them the opportunity to face death as a natural and necessary event in life that can be managed with sensitivity, generosity and serenity. Participants’ responses in the qualitative part of the study revealed that they had discovered a deeper meaning in their lives and developed better coping skills to deal with loss, empowering them with more confidence to talk about the subject. In addition, some participants reported a change in their death representations from death as total annihilation to death as a passage, suggesting that they had gotten closer to a spiritual and transcendental dimension. For some, spirituality was a positive discovery, while for others, it simply confirmed their faith. From this, we conclude that it is important and desirable to invest in new DE programs, as they have proven to be effective in addressing and reducing the denial of death.

One limitation of this study, common to many DE programs carried out in schools, is that it was not possible to randomise the DE and NoDE groups, as only those classes that were willing to participate were involved in the study. We recommend that future studies explore extracurricular DE courses to overcome this issue. Furthermore, it would be interesting to investigate whether the positive effects of the course persisted through a follow-up study, and to extend this investigation to teachers and hospice professionals.

## Supplementary Information

**Additional file 1: Table A.** Analysis of the differences between females and males. **Table B.** Analysis of the differences between believers and non-believers in God.

## Data Availability

The datasets used and/or analysed during the current study are available from the corresponding author on reasonable request.

## Abbreviations

DE: Death Education
NoDE: No Death Education
TDRS: Testoni Death Representation Scale
TAS: Toronto Alexithymia Scale
PMP: Personal Meaning Profile
SOI: Spiritual Orientation Inventory
