# Supplementary material for: The hospice as a learning space: a death education intervention with a group of adolescents
Source: BMC Palliat Care. 2021 Apr 7;20:54. doi: 10.1186/s12904-021-00747-w (PMC8028247; doi:10.1186/s12904-021-00747-w)
Supplement: Supplementary file 1 — Additional file 1: Table A. Analysis of the differences between females and males. Table B. Analysis of the differences between believers and non-believers in God. [file 12904_2021_747_MOESM1_ESM.docx]

**Table A - Analysis of the differences between females and males**

| Scale | **Females (N=110)** | | **Males (N=53)** | | **Results of test t** | |
| --- | --- | --- | --- | --- | --- | --- |
|  | M | SD | M | SD | t (df=161) | p-value |
| TDRS | 3.24 | 0.93 | 3.31 | 0.94 | -0.42 | .674 |
| TAS_Tot | 52.35 | 10.77 | 48.34 | 10.76 | 2.23 | .027 |
| Factor 1 | 2.76 | 0.86 | 2.24 | 0.82 | 3.66 | .000 |
| Factor 2 | 3.07 | 0.93 | 2.69 | 0.84 | 2.57 | .011 |
| Factor 3 | 2.21 | 0.56 | 2.41 | 0.62 | -2.01 | .046 |
| PMP_Tot | 4.54 | 0.69 | 4.69 | 0.66 | -1.26 | .209 |
| ACH | 4.88 | 0.97 | 5.20 | 0.87 | -2.01 | .046 |
| RLT | 5.02 | 0.89 | 5.11 | 0.91 | -0.58 | .566 |
| RLG | 3.64 | 1.09 | 3.66 | 1.39 | -0.13 | .895 |
| ST | 4.36 | 0.98 | 4.73 | 1.15 | -2.18 | .031 |
| SA | 4.32 | 1.10 | 4.60 | 0.89 | -1.58 | .115 |
| INT | 4.84 | 1.21 | 4.65 | 1.22 | 0.96 | .337 |
| FT | 4.75 | 0.96 | 4.87 | 0.93 | -0.77 | .441 |
| SOI | 3.37 | 0.56 | 3.20 | 0.75 | 1.62 | .107 |

**Table B - Analysis of the differences between believers and non-believers in God**

| Scale | **Believers in God (N=91)** | | **No Believers in God (N=72)** | | **Results of test t** | |
| --- | --- | --- | --- | --- | --- | --- |
|  | M | SD | M | SD | t (df=161) | p-value |
| TDRS | 2.97 | 0.88 | 3.64 | 0.86 | -4.89 | <.001 |
| TAS_Tot | 50.98 | 11.16 | 51.13 | 10.65 | -0.08 | .934 |
| Factor 1 | 2.58 | 0.84 | 2.60 | 0.94 | -0.12 | .904 |
| Factor 2 | 2.97 | 0.85 | 2.93 | 1.00 | 0.29 | .773 |
| Factor 3 | 2.26 | 0.60 | 2.29 | 0.57 | -0.31 | .755 |
| PMP_Tot | 4.68 | 0.64 | 4.48 | 0.73 | 1.86 | .065 |
| ACH | 4.92 | 0.99 | 5.06 | 0.89 | -0.96 | .339 |
| RLT | 4.97 | 0.86 | 5.14 | 0.94 | -1.21 | .230 |
| RLG | 4.24 | 1.10 | 2.90 | 0.83 | 8.61 | <.001 |
| ST | 4.52 | 1.05 | 4.42 | 1.05 | 0.61 | .541 |
| SA | 4.45 | 0.94 | 4.37 | 1.16 | 0.46 | .649 |
| INT | 4.88 | 1.09 | 4.65 | 1.34 | 1.22 | .225 |
| FT | 4.76 | 0.91 | 4.81 | 1.00 | -0.33 | .745 |
| SOI | 3.48 | 0.55 | 3.10 | 0.66 | 4.00 | <.001 |
